# Abdominal and hip fat percentages are associated with fragility fractures: evidence from a large cross-sectional study

**DOI:** 10.1038/s41598-025-07648-5

**Published:** 2025-07-02

**Authors:** Hamzah Amin, Muhammed Aqib Khan, Marwan Bukhari

**Affiliations:** 1 Lancaster University Medical School, Sir John Fisher Drive, Lancaster, LA1 4YW UK; 2 Royal Lancaster Infirmary, Lancaster, UK

**Keywords:** Regional adiposity, Fragility fractures, Hip fracture, Bone, Ageing, Rheumatology, Bone, Cells, Adipocytes

## Abstract

The distribution of body fat may influence fracture risk, yet the association between DXA-derived regional fat and fragility fractures remains unclear. We analysed 36,235 patients undergoing lumbar spine and femoral DXA scans from June 2004 to February 2024, categorizing abdominal, left hip, and right hip fat percentages into tertiles (low, medium and high). Multivariable logistic regression models were fit with the dependant variable being hip or any fragility fracture and the independent variable being the binary regional body composition groups. All results were adjusted for FRAX clinical factors where we found that the lowest tertile of abdominal fat was associated with reduced odds of any fracture (OR 0.78, 95% CI 0.74–0.82), while the highest tertile was linked to increased odds (OR 1.22, 95% CI 1.17–1.29) but was not associated with hip fracture. For left hip fat, the lowest tertile was linked to decreased odds of any fracture (OR 0.61, 95% CI 0.58–0.64), and the highest tertile showed increased odds of any fracture (OR 1.57, 95% CI 1.50–1.65) and hip fracture (OR 1.32, 95% CI 1.07–1.63). Similarly, the right hip fat lowest tertile was associated with reduced odds of hip fracture (OR 0.74, 95% CI 0.58–0.93) and any fracture (OR 0.62, 95% CI 0.59–0.66), while the highest tertile was linked to increased odds of hip fracture (OR 1.68, 95% CI 1.37–2.07) and any fracture (OR 1.59, 95% CI 1.52–1.67). Our results suggest DXA derived regional body composition may be a novel predictor of both hip and any fragility fractures with further research needed to validate our findings.

## Introduction

Osteoporosis is defined as a reduction in bone mineral density (BMD) 2.5 standard deviations below that of a healthy young adult population^1^. Clinically, it remains asymptomatic until a fragility fracture occurs, hence early identification and stratification of at risk populations remains a central part of osteoporosis management^1^. Fracture risk calculators such as FRAX and QFracture are both commonly used in clinical practice and combine multiple known risk factors for fracture including bone mineral density. FRAX has recently begun working on a updated calculator called FRAXPlus which now includes additional risk factors including type 1 diabetes, and previous osteoporotic fracture as an example^2^. However advanced body composition techniques beyond body mass index (BMI) have yet to be integrated into either of these tools to improve the accuracy of their calculations^2^.

BMI is defined as weight over height squared and is very easy to calculate in clinical practice. High BMI has been associated with increases in BMD and a decrease in fracture risk and vice versa for low BMI^3^. However in fracture risk calculators BMI is largely dependent on BMD and its predictive value on its own is limited including in obese populations who tend to fracture at normal BMDs^3^. Furthermore, BMI is a crude measure that does not reflect true body composition (fat mass versus lean mass) and is inadequate for detecting body composition disorders like sarcopenia, sarcopenic obesity, and osteosarcopenia, all of which are increasingly recognized as important factors in fracture risk^4^. Hence approaches beyond BMI that are sensitive to changes in body composition are needed to improve the accuracy of fracture risk calculators.

While high BMI is generally associated with increased bone density, the same does not hold true for high levels of adipose tissue^5^. Research suggests that excessive adipose tissue exerts complex and often detrimental effects on bone health. High levels of adipose tissue have been linked to chronic low-grade inflammation^6^, elevated adipokine levels^7^, and vitamin D deficiency—all of which impair bone quality and increase fracture risk^8^. Furthermore the biomechanical effects of high levels of adiposity have been associated with an increased risk of falls^9^, due to shifting the body’s centre of mass, which can increase postural instability and predispose individuals to falls and fractures^10^. In addition to these mechanisms between adiposity and fragility fractures the distribution of adipose tissue is also said to influence both bone density and fracture risk^11^.

Two primary clinical phenotypes based on fat distribution are often discussed in the literature: the “apple” phenotype, characterized by elevated levels of intra-abdominal (visceral) fat and the “pear” phenotype which is associated with peripheral fat storage in areas such as the hips and thighs^12^. Intrabdominal Visceral fat has been shown to correlate with reduced bone density and an increased risk of fracture, with many studies employing waist-to-hip ratios and waist circumference as proxies for fat distribution^13^. However, when studies have used dual-energy X-ray absorptiometry (DXA) derived abdominal fat mass this has shown mixed results^14,15^. Conversely, while gynoid or peripheral obesity may be linked to a higher risk of falls^16^, its direct relationship with fracture risk remains to be clearly established. Hence further research is needed to clearly elaborate how DXA derived regional fat distribution affects fracture risk.

DXA is a highly accurate way of measuring body composition, with a full body DXA scan being the gold standard way of measuring body composition^17^, though this is not routinely performed in clinical practice^18^. However many patients who undergo diagnostic work up for osteoporosis will have bilateral femoral and lumbar spine DXA scans^19^ which also gives us body composition data at said sites^20^, though this information is not reported on the scan report, though stored in the systems database. Hence using this data could be a novel way of considering regional body fat data in fracture risk calculators beyond simple measures such as the waist-to-hip ratio.

## Aims

The aim of this study is to assesses whether DXA derived regional adiposity is associated with hip or any major fragility fracture.

## Methods

### Data collection

Patients were referred from primary and secondary care to our DXA scanner in the northwest of England between June 2004 and February 2024. Our regional scanner serves a population of 365,000 patients and sees roughly 2,300 patients per year. Two scanners have been used during this period: the GE Lunar Prodigy (2004–2019) and, more recently, the GE Lunar iDXA (2019–present). Cross-calibration was conducted at the time of system transition using scan phantoms to ensure continuity and accuracy of measurements. Routine quality control is performed weekly also using scan phantoms to maintain measurement precision. All referrals are vetted by staff prior to acceptance.

Typically, patients undergo a combined scan of the lumbar spine and bilateral femoral regions, which is standard practice at our regional scanner. The lumbar spine scan includes the L1–L4 region while the femoral scans included the femoral head, neck, wards triangle, trochanters, and part of the shaft. These scans provided both bone density and compositional data for these sites. Bone mineral density was reported as a T-score. For compositional data, the DXA scan measured lean mass, fat mass, and calculated the local fat percentage at each site.

Fracture history was obtained by the technician to determine whether the patient had experienced a fragility fracture, defined as a fracture resulting from a fall from standing height or less over the age of 50. The technician verified both the mechanism and anatomical site of each reported fracture prior to data entry.

Demographic and FRAX risk factors were collected via a standardised questionnaire. Patients were asked regarding the presence of fracture risk factors which was then inputted by the technician into the system. Risk factors included whether the patient was a current smoker, was currently taking glucocorticoids (defined as > 5 mg per day of prednisolone or equivalent for > 3 months at the time of the appointment as per FRAX), or consumed excess alcohol (> 3 units per day), had rheumatoid arthritis or had a parental history of fragility fracture.

All data were stored in a SQL server where data was then extracted and synthesised to form our database for analysis. Full ethical approval for pseudonymized data extraction in the absence of informed consent was obtained from the local ethics committee (NRES Committee Northwest Preston, project number 14/NW/1136).

### Data analysis

Only scanned patients who underwent combined bilateral femoral and abdominal scans were included in the analysis. Baseline characteristics were first compared between the fractured and non-fractured populations. Comparative statistics were used including the student’s t-test for normally distributed continuous data and Pearson’s chi-squared test for categorical data.

To allow comparison, fat percentages at the abdomen (FPA), left hip (FPLH), and right hip (FPRH) were first stratified into tertiles, with tertile 1 representing the lowest fat percentage and tertile 3 the highest. Next, binary groups were created from the tertiles; for example, patients in the lowest tertile of abdominal fat were coded as a one while the rest of the population was coded as a zero. This was repeated for all other tertiles at each anatomical sites creating 9 binary groups in total, which was the foundation of our statistical analysis. We decided to use this approach versus using continuous variables for fat percentages to allow for ease of interpretation as well as effectively handling any non-linearity which may have been present.

Each of the nine body composition groups was then used as the independent variables in separate multivariate logistic regression models to assess associations with femoral (hip) fractures and with any fragility fracture. Separate models were employed to avoid collinearity and ensure numerical stability. The definition of ‘any fragility fracture’ encompassed major osteoporotic sites (spine, humerus, wrist), as well as tibial, fibular, and ankle fractures. Hip fractures were analysed independently due to their disproportionately high associated morbidity and mortality^21^.

All models were adjusted for the same confounders: gender, age, current smoking, excessive alcohol use (> 3 units/day, per FRAX), steroid therapy (> 5 mg/day for > 3 months), rheumatoid arthritis, personal and family history of fragility fractures, and left femoral total T-score. A Bonferroni-corrected p-value of 0.017 (0.05/3) was applied to account for the three groups analysed per anatomical site. STATA version 18 was used in our analysis.

## Results

### Demographics

A total of 36,235 patients were included in the analysis, of whom 14,342 reported a fragility fracture including 383 femoral fractures. The mean (SD) age of all patients was 65.7 (12.9) years, height was 162.3 (8.6) cm, weight was 71.6 (16.6) kg, and BMI was 27.1 (5.7) kg/m^2^. The mean left femoral T-score for all patients was − 1.0 (1.3).

Patients reporting fragility fractures were older (66.0 vs. 65.3 years, *P* < 0.001) and more often female (84.0% vs. 83.0%, *P* = 0.013). They were slightly shorter (162.1 vs. 162.4 cm, *P* = 0.003), while weight and BMI did not differ significantly. Smoking rates were comparable between groups, but higher alcohol consumption (> 3 units/day) was more common among those with fractures (8.0% vs. 6.6%, *P* < 0.001). Glucocorticoid use (6.1% vs. 11.6%, *P* < 0.001) and rheumatoid arthritis (5.5% vs. 6.9%, *P* < 0.001) were less frequent in the fracture group. Family history of fracture showed no significant difference. DXA T-scores were significantly lower in fractured patients at the left femur (− 1.2 vs. − 0.75), right femur (− 1.2 vs. − 0.77), and lumbar spine (− 0.8 vs. − 0.2) (all *P* < 0.001). These results can be seen in Table 1.

**Table 1:** Compositional factors compared between fractured and non-fractured patients.

Risk factor	Fracture (N = 14,342, 39.6%)	Non-fracture (N = 21,893, 60.4%)	*P*-value
Demographics			
Age (years)	66.0 (12.5)	65.3 (13.0)	< 0.001
Gender	12,046 (84.0%) (female)	18,170 (83.0%) (female)	0.013
Anthropometric/body composition			
Height (cm)	162.1 cm (8.5)	162.4 cm (8.7)	0.003
Weight (kg)	71.5 kg (16.2)	71.7 kg (16.5)	0.170
Body Mass Index (kg/m^2^)	27.1 kg/m^2^ (5.5)	27.1 kg/m^2^ (5.5)	0.741
Fat percentage at the abdomen	32.1% (10.5)	31.4% (10.8)	0.001
Fat percentage at the left femur	31.6% (6.9)	29.7% (7.0)	0.001
Fat percentage at the right femur	31.0% (6.9)	29.2% (6.9)	0.001
Lifestyle factors			
Current smoker (n = 3885; 10.7%)	1500 (10.5%)	2385 (10.8%)	0.190
Alcohol use* (> 3 units per day) (n = 2605; 7.2%)	1150 (8.0%)	1455 (6.6%)	0.001
Medical history			
Glucocorticoid use (n = 4676; 12.9%)	880 (6.1%)	2530 (11.6%)	0.001
Rheumatoid arthritis (n = 2301; 6.4%)	782 (5.5%)	1519 (6.9%)	0.001
Family history of a fracture (n = 7943; 21.9%)	3151 (22.0%)	4783 (21.8%)	0.781
DXA outcomes			
Left femoral (total) T-score	− 1.2 (1.21)	− 0.75 (1.30)	0.001
Right femoral (total) T-score	− 1.2 (1.20)	− 0.77 (0.79)	0.001
Lumbar spine T-score	− 0.8 (1.7)	− 0.2 (1.7)	0.001

The mean fat percentages in the full cohort were 30.45% (SD 7.01) at the left femur, 29.93% (6.93) at the right femur, and 31.66% (10.72) at the abdomen. Patients with fractures had significantly higher fat percentages at the left femur (31.6% vs. 29.7%), right femur (31.0% vs. 29.2%), and abdomen (32.1% vs. 31.4%) (all *P* < 0.001). Tertiles for fat distribution were defined as abdominal fat ≤ 27.42%, 27.43–37.44%, and ≥ 37.44%; left femoral fat ≤ 27.34%, 27.35–33.40%, and ≥ 33.40%; right femoral fat ≤ 26.83%, 26.84–32.79%, and ≥ 32.79%. These cutoffs can also be seen in Table 2.

**Table 2:** Tertile cutoffs for fat percentage across anatomical sites.

	Tertile 1 (%)	Tertile 2 (%)	Tertile 3 (%)
Abdominal fat percentage	≤ 26.83	26.83–32.79	≥ 32.79
Left femur fat percentage	≤ 27.34	27.34–33.4	≥ 33.40
Right femur fat percentage	≤ 27.42	27.43–37.44	≥ 37.44

### Primary analysis results

Our primary analysis revealed significant associations between regional fat distribution and the odds of fractures. For abdominal fat, patients in Tertile 1 (lowest) had significantly lower odds of experiencing any fragility fractures (OR 0.78, 95% CI 0.74–0.82, *P* < 0.001), while those in Tertile 3 (highest) had higher odds (OR 1.22, 95% CI 1.17–1.29, *P* < 0.001). This relationship between lower fat levels and reduced odds of any fracture was also observed for the left femoral region (OR 0.61, 95% CI 0.58–0.64, *P* < 0.001) and right femoral region (OR 0.62, 95% CI 0.59–0.66, *P* < 0.001). Conversely, increased odds were seen in the highest tertile of regional fat and any fragility fracture for both the left femoral (OR 1.57, 95% CI 1.50–1.65, *P* < 0.001) and right femoral fat percentages (OR 1.59, 95% CI 1.52–1.67, *P* < 0.001). These results can be seen in Tables 3, 4 and 5.

**Table 3:** Association between tertiles of abdominal fat and odds of hip or any fragility fracture (reporting odds ratios (95% CI)).

	Tertile 1—abdominal	Tertile 2—abdominal	Tertile 3—abdominal
Multivariable regression analysis looking at the relationship between tertiles of abdominal fat percentages and hip/any fractures			
Hip fracture	0.83 (0.66, 1.04)	1.18 (0.95, 1.46)	1.02 (0.81, 1.27)
Any fracture	**0.78 (0.74, 0.82)**	1.04 (0.99, 1.09)	*1.22 (1.17, 1.29)*

All results are adjusted for fracture risk factors. Bold value indicates a statistically significant decrease in odds, italic value indicates a statistically significant increase in odds, and roman values indicate no significant association.

**Table 4:** Association between tertiles of left hip fat and odds of hip or any fragility fracture (reporting odds ratios (95% CI)).

	Tertile 1—left hip	Tertile 2—left hip	Tertile 3—left hip
Multivariable regression analysis looking at the relationship between tertiles of left hip fat percentages and hip/any fractures			
Hip fracture	0.83 (0.66, 1.04)	0.89 (0.71, 1.11)	*1.32 (1.07, 1.63)*
Any fracture	**0.61 (0.58, 0.64)**	1.03 (0.98, 1.08)	*1.57 (1.50, 1.65)*

All results are adjusted for fracture risk factors. Green value indicates a statistically significant decrease in odds, italic values indicate a statistically significant increase in odds, and roman values indicate no significant association.

**Table 5:** Association between tertiles of right hip fat and odds of hip or any fragility fracture (reporting odds ratios (95% CI)).

	Tertille 1—Right hip	Tertile 2—Right hip	Tertile 3—Right hip
Multivariable regression analysis looking at the relationship between tertiles of right hip fat percentages and hip/any fractures			
Hip fracture	**0.74 (0.58, 0.93)**	0.75 (0.59, 0.94)	*1.68 (1.37, 2.07)*
Any fracture	**0.62 (0.59, 0.66)**	1.0 (0.95, 1.05)	*1.59 (1.52, 1.67)*

All results are adjusted for fracture risk factors. Bold values indicate a statistically significant decrease in odds, italic values indicate a statistically significant increase in odds, and roman values indicate no significant association.

Interestingly, for hip fractures, only high fat percentages (tertile 3) in the femoral regions were associated with increased odds of hip fracture. Specifically, elevated left femoral fat (OR 1.32, 95% CI 1.07–1.63, *P* = 0.010) and right femoral fat (OR 1.68, 95% CI 1.37–2.07, *P* < 0.001) were linked to higher odds of hip fractures. The lowest tertile of right femoral fat was associated with decreased odds of hip fracture (OR 0.74, 95% CI 0.58–0.93, *P* = 0.011). No other significant findings were seen for between regional body composition and hip fractures. These results can also be seen in Tables 3, 4 and 5.

## Discussion

To our knowledge, this study is one of the first to explore how hip fat percentage affects fragility fractures. While previous studies often look at abdominal fat^13^, the hip sites have seldom been explored in relation to fragility fractures. Our findings provide further insight into the role of regional fat deposition and its potential contribution to fracture risk.

Our analysis revealed compositional differences between fractured and non-fractured populations, with height being the only significantly different anthropometric variable—fractured patients were shorter. Surprisingly, neither weight nor BMI differed significantly between the groups. This contrasts with previous research that has highlighted associations between lower weight^22,23^, reduced BMI^3^, and fragility fractures. However, despite the lack of significance in weight and BMI, regional fat was notably higher in the fracture group, though the difference was relatively small in our univariate analysis. However, we hypothesise this could be the case given most of our patient group were homogenous given the fact they were all at-risk for osteoporosis. More heterogenous populations may have had larger difference in regional body composition between those reporting a fragility fracture versus those not reporting one. However this does however underscore a key limitation of BMI, which may not adequately capture body composition differences between fractured, and none fractured individuals that are important to fracture risk. This is especially problematic in elderly at risk populations who often undergo complex compositional changes including increasing adiposity and a reduction in muscle mass^24^.

In our primary analysis for any fragility fracture, we found that patients in the highest tertile of fat at all anatomical sites had significantly increased odds of any fractures, with the peripheral regions (hips) showing higher odds than centrally. Conversely, patients in the lowest tertile of fat had reduced odds of any fractures. Importantly, these associations were independent of known confounders such as age, BMI, smoking status, alcohol consumption, and BMD, further strengthening the robustness of our findings. Our findings could suggest that regional body fat could be an important independent factor associated with fragility fractures.

Several biological mechanisms could explain these results. Higher abdominal fat may alter a person’s centre of gravity^10^, increasing the risk of falls and fractures. This is supported by previous research showing that patients with high abdominal fat are more likely to be dynapenic and have an increased falls risk^25–27^. Hence our abdominal fat measurement may have captured this association.

Regarding hip fat as a predictor of any fracture it could be possible that patients with high levels of hip fat could have a reduction in the quality of gluteal muscles due to fatty infiltration^28^ which could impair both balance and stability^29^. Conversely, individuals with lower gluteal fat levels may have higher lean muscle mass, providing better physical control and protection against fractures. These findings emphasize the need for further research to better understand how fat distribution impacts muscle quality and affects both falls and subsequent fracture risk.

When focusing specifically on hip fractures, our analysis found a strong association between high hip fat and hip fracture. Interestingly there was no association found between abdominal fat and hip fractures. This does support the study conducted by Nguyen et al. which also looked at DXA derived abdominal fat as a predictor of hip fracture and concluded the relationship was likely to be modest at best^14^. We agree with this and believe that measuring hip fat may be a better way of predicting hip fracture. This could be the case as high hip fat would precipitate instability due to a reduction in gluteal muscle quality with high fat potentially not providing the cushioning effect which is often described in the literature^30^. Further research is needed with prospective cohorts to corroborate the association we have seen.

Given that high hip fat may mean a reciprocal decrease in muscle mass locally^31^, it would be interesting to know whether these patients are also sarcopenic or sarcopenic obese. This approach could be an indirect way of evaluating these patients for both body composition disorders in fracture risk calculators. Further research correlating regional body fat composition to accepted definitions of sarcopenia would be useful in elucidating its diagnostic utility in identifying these compositional disorders.

Additionally, it remains important to assess whether fat percentage from both hips is required to predict fragility fractures, or if a single hip measurement suffices. Prior studies suggest lower limb dominance affects BMD, with the non-dominant hip typically showing lower BMD values^32^. In our cohort, the difference in fat percentage between left and right femurs among fractured vs non-fractured individuals was modest (0.6%), with slightly higher fat on the left side. Furthermore, given the 95% confidence intervals in our multivariable models also overlapped when assessing both any fracture and hip fractures, this suggests that the odds from both hip were relatively similar. However, given that leg dominance influences BMD^32^, it may be hypothesised that it could affect regional body composition and fracture risk, though further research is needed to confirm this. Further research is ultimately needed to clarify the influence of hip dominance and regional compositional asymmetries on fracture risk.

We also believe that our findings also challenge the conventional “apple” (abdominal fat) and “pear” (hip fat) understanding of body composition and associated measurements including the waist to hip ratio (WHR) and waist circumference (WC). While previous studies have associated higher intra-abdominal fat with increased fracture risk^13^, these studies often rely on indirect measures, such as the WC or WHR, which generally suggest that peripheral fat may be protective. In contrast, our results imply that excessive peripheral fat, particularly in older adults, may be associated with fragility fractures including hip fractures specifically. Further research is warranted to explore the relationship between regional fat, waist circumference, waist-to-hip ratio, and whether direct measurements of regional fat provide a more accurate reflection of fat distribution and its clinical implications including fracture risk.

One of the key unresolved questions raised by our study is whether high regional fat precedes fractures, or if fractures themselves lead to compositional changes over time. Due to the cross-sectional design of our study, we cannot establish causality, but we hypothesize that increased fat may contribute both to initial fractures and to subsequent fractures. Longitudinal studies are needed to clarify the directionality of this relationship and to determine whether regional fat could serve as an early marker of fracture risk.

Finally, while our study used arbitrary cut-offs for regional body fat, generating reference data for normal ranges of regional fat percentages could improve fracture risk prediction in this population. Establishing these normative data would allow for more accurate identification of patients at risk for fractures.

## Strengths and limitations

This study is limited by its cross-sectional design, which prevents the evaluation of temporal relationships between regional fat distribution and fracture risk. Consequently, it remains unclear whether increased adiposity precedes fractures or results from them. Furthermore, the study population was also relatively homogeneous, comprising primarily Caucasian individuals at elevated risk for osteoporosis, which may limit the generalizability of the findings to the general population. Additionally, some participants may have been referred following a fracture event, introducing potential confounding by indication. We were also unable to determine the duration of exposure to fracture-related risk factors, which may have influenced the observed associations. Finally, the study is subject to potential recall and sampling biases, which could affect the validity of the results.

Our study is strengthened by large number of patient data we analysed, as well as the utilisation of routinely collected health data for regional body composition, which would not add any extra burden which mimics current clinical practice. All our patients underwent DXA scanning, the gold standard for both BMD assessment as well as fat mass data. Furthermore, we have analysed our results by looking at the more clinically useful outcome of fragility fractures as opposed to BMD.

## Conclusions

Our study demonstrated that there was no statistically significant difference in BMI between fractured and non-fractured patients; however, patients with fractures exhibited slightly higher adiposity across all regional sites we analysed. In terms of fragility fractures, low regional adiposity was associated with decreased odds of any fracture, while high regional adiposity was associated with increased odds of any fracture. Specifically, for hip fractures, only elevated left and right hip fat were associated with an increased odds of fracture. We hypothesize that regional fat, particularly at the hips, may serve as a novel predictor of fracture risk and could be biomechanically relevant with its relationship to falls risk and instability. However, further research is needed to validate our findings and hypotheses.

## Data Availability

Data can be made available upon reasonable request to the corresponding author.

## References

[1] Akkawi, I. & Zmerly, H. Osteoporosis: Current concepts. *Joints* **6**(2), 122–127 (2018). 30051110 10.1055/s-0038-1660790 PMC6059859

[2] El Miedany, Y. FRAX: Re-adjust or re-think. *Arch. Osteoporos.* **15**(1), 150 (2020). 32989561 10.1007/s11657-020-00827-z PMC7522068

[3] De Laet, C. et al. Body mass index as a predictor of fracture risk: A meta-analysis. *Osteoporos. Int.* **16**(11), 1330–1338 (2005). 15928804 10.1007/s00198-005-1863-y

[4] Curtis, M., Swan, L., Fox, R., Warters, A. & O’Sullivan, M. Associations between body mass index and probable sarcopenia in community-dwelling older adults. *Nutrients* **15**(6), 1505 (2023). 36986233 10.3390/nu15061505 PMC10059806

[5] Jain, R. K. & Vokes, T. Visceral adipose tissue is negatively associated with bone mineral density in NHANES 2011–2018. *J. Endocr. Soc.* **7**(4), 008 (2023). 10.1210/jendso/bvad008 PMC9922944 36793478

[6] Lumeng, C. N. & Saltiel, A. R. Inflammatory links between obesity and metabolic disease. *J. Clin. Invest.* **121**(6), 2111–2117 (2011). 21633179 10.1172/JCI57132 PMC3104776

[7] Hou, J. et al. Obesity and bone health: A complex link. *Front. Cell Dev. Biol.* **8**, 600181 (2020). 33409277 10.3389/fcell.2020.600181 PMC7779553

[8] Chen, R. & Armamento-Villareal, R. Obesity and skeletal fragility. *J. Clin. Endocrinol. Metab.* **109**(2), e466–e477 (2024). 37440585 10.1210/clinem/dgad415 PMC10795939

[9] Neri, S., Oliveira, J., Dario, A., Lima, R. & Tiedemann, A. Does obesity increase the risk and severity of falls in people aged 60 years and older? A systematic review and meta-analysis of observational studies. *J. Gerontol. A Biol. Sci. Med. Sci.* **75**(5), 952–960 (2020). 31750880 10.1093/gerona/glz272

[10] Del Porto, H., Smith, D. & Reed-Jones, R. Biomechanical effects of obesity on balance. *Int. J. Exerc. Sci.* **5**(4), 301–320 (2012).

[11] Zhu, X. W. et al. General and abdominal obesity operate differently as influencing factors of fracture risk in old adults. *iScience* **25**(6), 104466 (2022). 35677640 10.1016/j.isci.2022.104466 PMC9167983

[12] Alser, M. & Elrayess, M. A. From an apple to a pear: Moving fat around for reversing insulin resistance. *Int. J. Environ. Res. Public Health* **19**, 21 (2022). 10.3390/ijerph192114251 PMC9659102 36361131

[13] Sadeghi, O., Saneei, P., Nasiri, M., Larijani, B. & Esmaillzadeh, A. Abdominal obesity and risk of hip fracture: A systematic review and meta-analysis of prospective studies. *Adv. Nutr.* **8**(5), 728–738 (2017). 28916573 10.3945/an.117.015545 PMC5593104

[14] Nguyen, N. D., Pongchaiyakul, C., Center, J. R., Eisman, J. A. & Nguyen, T. V. Abdominal fat and hip fracture risk in the elderly: The Dubbo Osteoporosis Epidemiology Study. *BMC Musculoskelet. Disord.* **6**(1), 11 (2005). 15727686 10.1186/1471-2474-6-11 PMC554111

[15] Machado, L. G. et al. Visceral fat measured by DXA is associated with increased risk of non-spine fractures in nonobese elderly women: A population-based prospective cohort analysis from the São Paulo Ageing & Health (SPAH) Study. *Osteoporos Int.* **27**(12), 3525–3533 (2016). 27351667 10.1007/s00198-016-3682-8

[16] Neri, S. G. Body fat distribution in obesity and the association with falls: A cohort study of Brazilian women aged 60 years and over. *Maturitas* **139**, 64–68 (2020). 32747043 10.1016/j.maturitas.2020.06.009

[17] Shepherd, J. A., Ng, B. K., Sommer, M. J. & Heymsfield, S. B. Body composition by DXA. *Bone* **104**, 101–105 (2017). 28625918 10.1016/j.bone.2017.06.010 PMC5659281

[18] Marra, M. et al. Assessment of body composition in health and disease using bioelectrical impedance analysis (BIA) and dual energy X-ray absorptiometry (DXA): A critical overview. *Contrast Media Mol. Imaging* **2019**, 3548284 (2019). 31275083 10.1155/2019/3548284 PMC6560329

[19] Slart, R. H. J. A. et al. Updated practice guideline for dual-energy X-ray absorptiometry (DXA). *Eur. J. Nucl. Med. Mol. Imaging* **52**(2), 539–563 (2025). 39316095 10.1007/s00259-024-06912-6 PMC11732917

[20] Covey, M. K., Berry, J. K. & Hacker, E. D. Regional body composition: Cross-calibration of DXA scanners–QDR4500W and Discovery Wi. *Obesity (Silver Spring)* **18**(3), 632–637 (2010). 19960003 10.1038/oby.2009.420

[21] Mariconda, M. et al. The determinants of mortality and morbidity during the year following fracture of the hip: A prospective study. *Bone Joint J.* **97**(3), 383–390 (2015). 25737523 10.1302/0301-620X.97B3.34504

[22] Ensrud, K. E. et al. Body size and hip fracture risk in older women: a prospective study. Study of Osteoporotic Fractures Research Group. *Am. J. Med.* **103**(4), 274–280 (1997). 9382119 10.1016/s0002-9343(97)00025-9

[23] Cummings, S. R. et al. Risk factors for hip fracture in white women. Study of Osteoporotic Fractures Research Group. *N. Engl. J. Med.* **332**(12), 767–773 (1995). 7862179 10.1056/NEJM199503233321202

[24] Ponti, F. et al. Aging and imaging assessment of body composition: From fat to facts. *Front. Endocrinol. (Lausanne)* **10**, 861 (2019). 31993018 10.3389/fendo.2019.00861 PMC6970947

[25] Máximo, R. O. et al. Abdominal obesity, dynapenia and dynapenic-abdominal obesity as factors associated with falls. *Braz. J. Phys. Ther.* **23**(6), 497–505 (2019). 30391361 10.1016/j.bjpt.2018.10.009 PMC6849078

[26] Lv, D., Shen, S. & Chen, X. Association between dynapenic abdominal obesity and fall risk in older adults. *Clin. Interv. Aging* **17**, 439–445 (2022). 35418747 10.2147/CIA.S347053 PMC9001023

[27] Smith, L. et al. Dynapenic abdominal obesity increases risk for falls among adults aged ≥ 50 years: A prospective analysis of the Irish longitudinal study on ageing. *J. Gerontol. A Biol. Sci. Med. Sci.* **79**, 1 (2024). 10.1093/gerona/glad104 37071490

[28] Pacicco, T. et al. Pelvic muscle size and myosteatosis: Relationship with age, gender, and obesity. *Indian J. Radiol. Imaging* **29**(2), 155–162 (2019). 31367086 10.4103/ijri.IJRI_414_18 PMC6639865

[29] Davis, D. L. et al. Gluteal muscle fatty infiltration, fall risk, and mobility limitation in older women with urinary incontinence: A pilot study. *Skeletal Radiol.* **52**(1), 47–55 (2023). 35896734 10.1007/s00256-022-04132-3 PMC10091062

[30] Pana, T. A. et al. Body fat percentage and the long-term risk of fractures. The EPIC-Norfolk prospective population cohort study. *Maturitas* **168**, 71–77 (2023). 36502648 10.1016/j.maturitas.2022.11.005 PMC7614563

[31] Sun, L. et al. Association between body fat and sarcopenia in older adults with type 2 diabetes mellitus: A cross-sectional study. *Front. Endocrinol. (Lausanne)* **14**, 1094075 (2023). 36777353 10.3389/fendo.2023.1094075 PMC9911832

[32] Schwarz, P., Jørgensen, N. R., Jensen, L. T. & Vestergaard, P. Bone mineral density difference between right and left hip during ageing. *Eur. Geriatr. Med.* **2**(2), 82–86 (2011).

